# Association of Preoperative Personality Traits with Six-Month Subjective Quality of Vision After Bilateral PanOptix Trifocal IOL Implantation: A Prospective Single-Center Study

**DOI:** 10.3390/vision10040070

**Published:** 2026-09-16

**Authors:** Anca Maria Rednik-Pușcă, Cristina Ariadna Nicula

**Affiliations:** 1 Oculens Clinic, 400012 Cluj-Napoca, Romania; 2 Department of Ophthalmology, “Iuliu Hațieganu” University of Medicine and Pharmacy, 400347 Cluj-Napoca, Romania; niculacristina65@yahoo.com

**Keywords:** trifocal intraocular lens, personality traits, Big Five, quality of vision, dysphotopsia

## Abstract

Purpose: To investigate the association between preoperative personality traits and subjective quality of vision six months after bilateral trifocal intraocular lens (IOL) implantation. Methods: This prospective, single-center study included 66 patients undergoing bilateral implantation of the AcrySof IQ PanOptix TFNT00 trifocal IOL. Personality traits were assessed preoperatively using the Mini-International Personality Item Pool (Mini-IPIP). Six months after surgery, participants completed the Quality of Vision (QoV) questionnaire. Associations between the five personality dimensions and global QoV score, as well as individual visual symptoms, were evaluated. Results: Postoperative visual acuity was good at all distances (mean binocular uncorrected visual acuity: 0.04 ± 0.05 logMAR at distance, 0.06 ± 0.05 logMAR at intermediate, and 0.08 ± 0.05 logMAR at near). All five personality dimensions were significantly associated with global QoV scores (all pFDR < 0.05): extraversion (ρ = −0.343), agreeableness (ρ = −0.378), conscientiousness (ρ = −0.344), and openness to experience (ρ = −0.318) were associated with better subjective quality of vision, whereas neuroticism was associated with worse QoV (ρ = 0.428). Neuroticism showed the strongest correlation with starburst perception (ρ = 0.603, *p* < 0.001), while agreeableness demonstrated the strongest negative association with blurred vision (ρ = −0.524, *p* < 0.001). Halo, glare, starburst, hazy vision, blurred vision, and fluctuation in vision were consistently associated with multiple personality dimensions. Conclusions: Personality traits are significantly associated with patients’ subjective perception of visual quality following bilateral trifocal IOL implantation. Assessing personality before surgery may help identify patients at greater risk of postoperative dissatisfaction and guide more personalized counseling for those considering premium intraocular lenses.

## 1. Introduction

Cataract surgery has become one of the most frequently performed procedures in ophthalmology, with excellent functional outcomes resulting from continuous advances in surgical techniques, biometric measurements, and intraocular lens (IOL) technology [1,2]. Over time, the goals of cataract surgery have stretched beyond visual rehabilitation alone, increasingly focusing on improving patients’ quality of life and reducing dependence on spectacles [1,2].

Modern premium intraocular lenses have been developed to provide functional vision at different distances while reducing spectacle dependence [3,4,5]. Multifocal intraocular lenses, particularly trifocal designs, provide good visual performance across distance, intermediate, and near ranges, making them a very good option for presbyopia correction [3,4,5]. Among multifocal designs, trifocal IOLs provide the most complete range of functional vision but are associated with a higher rate of photic phenomena, such as glare, halos, and starbursts, than non-diffractive extended depth-of-focus designs [5]. For the PanOptix platform specifically, pooled multinational data indicate high rates of spectacle independence [6] alongside a substantial prevalence of glare, halos, and starbursts, although most cases are mild [7].

Despite achieving good optical performance and excellent postoperative visual acuity, a considerable proportion of patients implanted with multifocal intraocular lenses report dissatisfaction related to their quality of vision [5,8,9,10]. Visual disturbances such as glare, halos, starbursts, blurred vision, or difficulties focusing may be present after surgery [5,8,9,10,11], and these complaints are not always explained by objective clinical findings [8,9,10,11,12].

Patient-reported outcome measure instruments, such as the Quality of Vision (QoV) questionnaire, have revealed significant interindividual variability in symptom perception, even among patients with similar objective results [8,9,11], suggesting that factors beyond optical performance influence how these disturbances are experienced [8,9,11,12].

Personality has been proposed as one such factor, impacting how patients perceive and tolerate visual symptoms [13]. The Five-Factor Model organizes personality into five relatively stable dimensions: extraversion, agreeableness, conscientiousness, neuroticism, and openness to experience. These traits shape how individuals perceive, interpret, and respond to physical symptoms and medical interventions, and such differences have been documented across a broad range of medical conditions [14,15,16,17]. Although previous studies have suggested a link between personality and satisfaction after multifocal IOL implantation, evidence remains limited by small, heterogeneous cohorts, differences in implanted IOL designs, and outcome measures focused primarily on overall satisfaction rather than individual visual symptoms [13,18,19].

Therefore, the aim of this study was to evaluate the association between personality traits and subjective quality of vision six months after bilateral trifocal intraocular lens implantation using the validated Quality of Vision (QoV) questionnaire, and to identify which specific visual symptoms are most strongly associated with each personality dimension. Given the correlational design and the large number of associations examined, this study should be considered exploratory and hypothesis-generating; the association between personality traits and global QoV was treated as the primary analysis, while symptom-level associations were considered exploratory.

## 2. Materials and Methods

### 2.1. Study Design and Participants

This prospective, single-center observational study was conducted at Oculens Eye Clinic, Cluj-Napoca, Romania. Patients underwent surgery between March 2025 and October 2025, with postoperative assessment completed six months after each patient’s second eye surgery. The study adhered to the tenets of the Declaration of Helsinki and received approval from the Institutional Ethics Committee of Oculens Eye Clinic (Approval No. 1/2025). Written informed consent was obtained from all participants prior to enrollment.

Consecutive patients undergoing bilateral cataract surgery with implantation of a trifocal intraocular lens were screened for eligibility, and those fulfilling all inclusion criteria were invited to participate in the study.

Inclusion criteria included age below 70 years, regular corneal topography confirmed by Scheimpflug tomography using the Pentacam system (OCULUS Optikgeräte GmbH, Wetzlar, Germany), corneal astigmatism below 1.00 diopter, and spherical equivalent between −6.00 and +6.00 diopters. Patients were also required to be willing and able to complete the study questionnaires and attend all follow-up visits.

Exclusion criteria included amblyopia, irregular corneal astigmatism, previous intraocular surgery, corneal opacities, clinically significant dry eye disease or ocular surface disease, glaucoma, retinal pathology including diabetic retinopathy and age-related macular degeneration, optic nerve disease, uveitis, neuro-ophthalmic disorders, and cognitive impairment limiting reliable questionnaire completion. Patients experiencing intraoperative or postoperative complications, a postoperative difference in visual acuity between eyes greater than one line, postoperative spherical equivalent outside ±1.00 D or incomplete six-month follow-up data were excluded from the final analysis.

### 2.2. Preoperative Assessment and Surgical Procedure

All patients underwent a comprehensive ophthalmological examination before surgery, including slit-lamp biomicroscopy, Goldmann applanation tonometry, manifest refraction, dilated fundus examination, ocular surface evaluation, corneal endothelial cell count by specular microscopy, and corneal tomography using the Pentacam system. Ocular biometry was performed using the IOLMaster 700 (Carl Zeiss Meditec, Jena, Germany), and intraocular lens power calculations were performed using the Barrett Universal II formula with emmetropia as the target refraction.

All procedures were performed by the same surgeon using standard phacoemulsification through a clear corneal incision followed by in-the-bag implantation of the intraocular lens. The same lens model, the AcrySof IQ PanOptix TFNT00 trifocal intraocular lens (Alcon Laboratories, Fort Worth, TX, USA), was implanted bilaterally in all patients. The interval between first-eye and second-eye surgery did not exceed four weeks.

### 2.3. Personality Assessment

Personality traits were assessed preoperatively using the Mini-International Personality Item Pool (Mini-IPIP), a validated 20-item questionnaire derived from the Five-Factor Model of personality [20,21]. The Mini-IPIP was selected for its compact format, reducing respondent burden while maintaining adequate psychometric properties, making it particularly suitable for clinical research settings [20,21]. Romanian Mini-IPIP items were adapted from the official Romanian IPIP translation [22].

The questionnaire evaluates five personality domains: extraversion (sociability and positive emotionality), agreeableness (cooperativeness and empathy), conscientiousness (organization and self-discipline), neuroticism (emotional instability and susceptibility to negative emotions), and openness to experience (curiosity, imagination, and preference for novelty) [23,24].

Each domain consists of four items scored on a five-point Likert scale ranging from 1 (“strongly disagree”) to 5 (“strongly agree”), resulting in possible scores ranging from 4 to 20 points for each trait. Negatively worded items were reverse-scored prior to analysis, with higher scores indicating greater expression of the respective personality trait [20].

### 2.4. Postoperative Clinical Assessment

Patients were evaluated six months after second-eye surgery, a time point commonly used to allow for meaningful neuroadaptation to postoperative visual phenomena, although adaptation may continue beyond this interval [25]. Monocular and binocular visual acuity were assessed under both uncorrected and corrected conditions at distance (4 m), intermediate (66 cm), and near (40 cm). Monocular acuity was assessed to confirm the absence of a clinically significant postoperative difference between the two eyes. Distance visual acuity was measured using standard logMAR charts, whereas intermediate and near visual acuities were evaluated using Radner reading charts and subsequently converted to logMAR notation for statistical analysis.

### 2.5. Quality of Vision Assessment

Subjective quality of vision was assessed six months after surgery using the Quality of Vision (QoV) questionnaire developed and validated by McAlinden et al. [8,11]. The questionnaire evaluates ten visual symptoms commonly reported after refractive lens surgery, including glare, halos, starbursts, hazy vision, blurred vision, distortion, double vision, fluctuation in vision, focusing difficulties, and depth perception difficulties.

For each visual symptom, patients reported its frequency, severity, and bothersome level in daily life using the original response format of the QoV questionnaire. For the purposes of this study, a composite score for each visual symptom was calculated by summing the frequency, severity, and bothersome scores (range: 0–9). A global QoV score was subsequently obtained by summing the composite scores across all ten visual symptoms (range: 0–90), with higher scores indicating a greater burden of visual symptoms and therefore poorer subjective quality of vision. To ensure consistent interpretation among participants, each visual symptom was accompanied by a verbal explanation and the corresponding example images from the original QoV questionnaire.

### 2.6. Statistical Analysis

Statistical analyses were performed using JASP software (version 0.18.3; JASP Team, University of Amsterdam, The Netherlands). Continuous variables were summarized as mean ± standard deviation (SD). Normality of data distribution was assessed using the Shapiro–Wilk test.

Given the non-normal distribution of most variables, associations between personality traits and subjective visual outcomes were analyzed using Spearman rank correlation coefficients (ρ). Correlations were calculated for both the global Quality of Vision (QoV) score and individual visual symptom scores. Corresponding 95% confidence intervals were calculated for all correlation coefficients.

A two-sided *p*-value of less than 0.05 was considered statistically significant. Given the exploratory nature of the analysis and the large number of correlations examined, *p*-values were additionally adjusted for multiple comparisons using the Benjamini–Hochberg false discovery rate (FDR) procedure, applied separately to the five global personality–QoV correlations and to the fifty trait-by-symptom correlations as two independent sets. As a sensitivity analysis, Spearman correlations between age and personality trait scores were calculated, and partial Spearman correlations between personality traits and global QoV scores, controlling for age, were performed to assess whether age confounded the observed associations. To evaluate potential multicollinearity among personality traits, pairwise Spearman correlations between all five personality dimensions were additionally examined. To further evaluate potential confounding, partial Spearman correlations between personality traits and global QoV were additionally calculated, controlling for sex and postoperative visual outcomes (spherical equivalent and binocular uncorrected visual acuity at distance, intermediate, and near).

## 3. Results

### 3.1. Study Population

**Figure 1:**
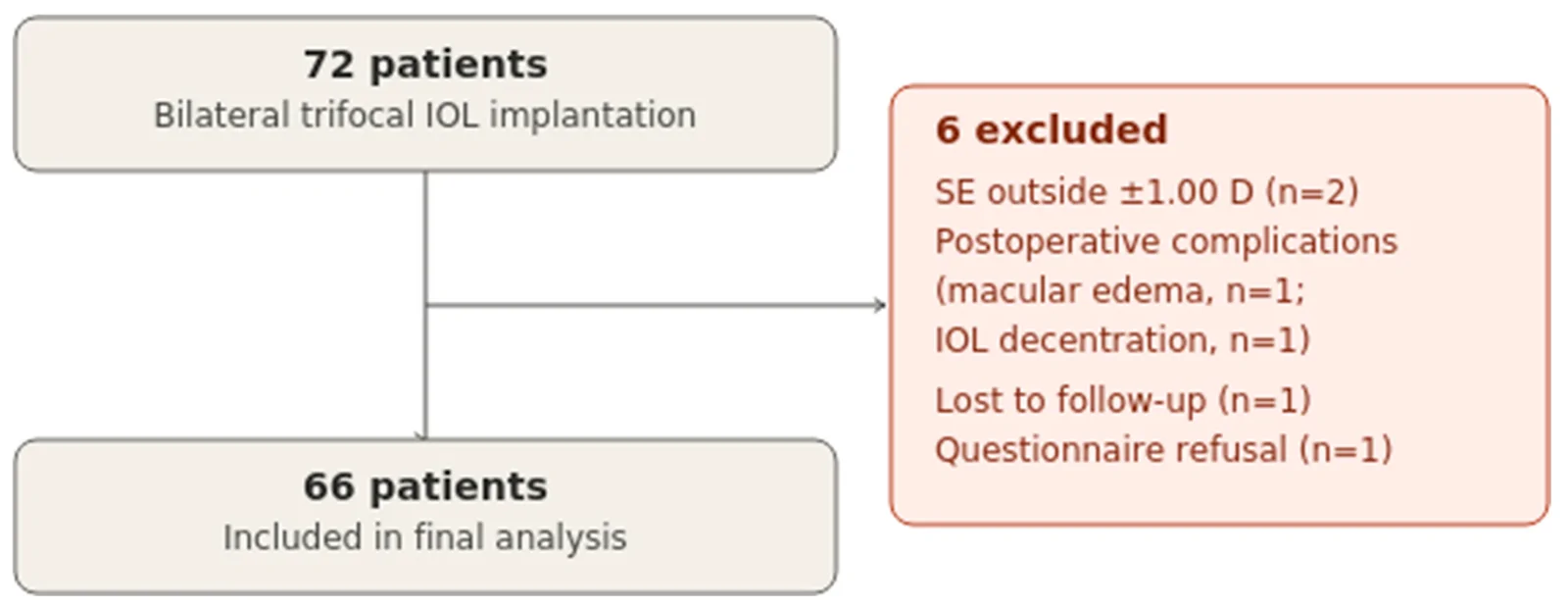
Participant flow diagram showing postoperative exclusions from the final analysis.

Of 72 patients who underwent bilateral implantation of the AcrySof IQ PanOptix TFNT00 trifocal intraocular lens, 6 were subsequently excluded from the final analysis, leaving 66 patients for analysis (Figure 1). The study population included 31 men (47.0%) and 35 women (53.0%), with a mean age of 62.70 ± 5.27 years.

Preoperatively, mean binocular uncorrected distance visual acuity was 0.52 ± 0.28 logMAR, improving to 0.33 ± 0.22 logMAR with correction. Mean uncorrected intermediate and near visual acuities were 0.66 ± 0.30 logMAR and 0.69 ± 0.30 logMAR, respectively, improving to 0.43 ± 0.24 logMAR and 0.42 ± 0.24 logMAR with correction.

Postoperative binocular visual acuity was high across all tested distances. Mean binocular uncorrected distance visual acuity (UDVA) was 0.04 ± 0.05 logMAR, while corrected distance visual acuity (CDVA) reached 0.01 ± 0.04 logMAR. Intermediate and near visual performance was similarly favorable, with mean uncorrected intermediate visual acuity (UIVA) of 0.06 ± 0.05 logMAR and corrected intermediate visual acuity (CIVA) of 0.03 ± 0.04 logMAR, and mean uncorrected near visual acuity (UNVA) of 0.08 ± 0.05 logMAR and corrected near visual acuity (CNVA) of 0.04 ± 0.04 logMAR.

Mean postoperative spherical equivalent was −0.08 ± 0.42 D (range −1.00 D to +1.00 D).

The mean QoV score was 34.91 ± 23.67 (median 40.0, IQR 13.0–48.0), while the mean scores for the five personality dimensions were 11.02 ± 4.53 (median 11.5, IQR 6.5–15.0) for extraversion, 14.23 ± 4.00 (median 14.0, IQR 11.0–18.0) for agreeableness, 13.53 ± 3.09 (median 14.0, IQR 11.0–15.0) for conscientiousness, 10.64 ± 3.30 (median 10.0, IQR 8.0–13.0) for neuroticism, and 13.47 ± 4.19 (median 14.0, IQR 10.5–17.8) for openness to experience.

### 3.2. Personality Traits and Global Quality of Vision

All five personality dimensions showed significant associations with global QoV scores, with correlation strengths ranging from weak to moderate (Table 1).

Neuroticism showed the strongest association with global QoV among all investigated traits, with higher neuroticism corresponding to poorer subjective quality of vision, whereas higher levels of extraversion, agreeableness, conscientiousness, and openness to experience were associated with better QoV.

### 3.3. Personality Traits and Individual Visual Symptoms

Personality traits were associated with several visual symptom domains, with the strength and pattern varying by trait (Table 2).

Extraversion was negatively correlated with most visual symptoms, most strongly with blurred vision and fluctuation in vision.

Agreeableness showed a similar pattern to extraversion, most strongly correlated with blurred vision.

Conscientiousness showed the broadest pattern of associations among the five traits, most strongly correlated with halo.

Neuroticism was the only trait associated with poorer rather than better visual outcomes. It correlated positively with most visual symptoms, most strongly with starburst perception—the strongest correlation found anywhere in the study.

Openness to experience correlated negatively with six symptom domains—glare, halos, starbursts, hazy vision, blurred vision, and fluctuation in vision—again with blurred vision showing the strongest association.

Following adjustment for multiple comparisons using the Benjamini–Hochberg false discovery rate procedure, all significant associations reported in the primary analysis remained statistically significant. Overall, higher levels of extraversion, agreeableness, conscientiousness, and openness to experience were consistently associated with fewer postoperative visual symptoms, whereas higher neuroticism scores were associated with a greater burden of dysphotopsias and poorer subjective quality of vision.

### 3.4. Sensitivity Analysis

Age showed weak but statistically significant correlations with conscientiousness (ρ = 0.249, *p* = 0.044) and neuroticism (ρ = −0.292, *p* = 0.017), but was not significantly correlated with global QoV (ρ = −0.077, *p* = 0.537) or with any individual visual symptom (all *p* > 0.05). To assess whether age confounded the observed personality–QoV associations, partial Spearman correlations controlling for age were calculated for all five personality traits. All associations stayed nearly the same and remained statistically significant (extraversion: partial ρ = −0.336, *p* = 0.006; agreeableness: partial ρ = −0.371, *p* = 0.002; conscientiousness: partial ρ = −0.336, *p* = 0.006; neuroticism: partial ρ = 0.425, *p* < 0.001; openness: partial ρ = −0.309, *p* = 0.012), indicating that age did not meaningfully confound the personality–QoV relationships identified in the primary analysis.

Extraversion, agreeableness, conscientiousness, and openness to experience were strongly and positively intercorrelated with one another (extraversion–agreeableness: ρ = 0.802; extraversion–conscientiousness: ρ = 0.513; extraversion–openness: ρ = 0.805; agreeableness–conscientiousness: ρ = 0.532; agreeableness–openness: ρ = 0.722; conscientiousness–openness: ρ = 0.658; all *p* < 0.001), whereas neuroticism showed weaker, negative correlations with each of the other four traits (neuroticism–extraversion: ρ = −0.249, *p* = 0.044; neuroticism–agreeableness: ρ = −0.353, *p* = 0.004; neuroticism–conscientiousness: ρ = −0.242, *p* = 0.051; neuroticism–openness: ρ = −0.121, *p* = 0.334).

Sex, postoperative spherical equivalent, and binocular uncorrected visual acuity at distance, intermediate, and near were not significantly associated with global QoV (all |ρ| ≤ 0.15, all *p* > 0.05). When personality–QoV correlations were additionally adjusted for these five variables simultaneously, all five associations remained similar in magnitude and statistically significant (extraversion: partial ρ = −0.337, *p* = 0.006; agreeableness: partial ρ = −0.365, *p* = 0.003; conscientiousness: partial ρ = −0.315, *p* = 0.010; neuroticism: partial ρ = 0.389, *p* = 0.001; openness: partial ρ = −0.296, *p* = 0.016).

## 4. Discussion

The present study investigated the association between personality traits and subjective quality of vision following bilateral implantation of a trifocal intraocular lens. The results show that personality characteristics are significantly associated with postoperative visual experience despite excellent objective visual outcomes. Higher levels of extraversion, agreeableness, conscientiousness, and openness to experience were associated with better subjective quality of vision and fewer reported visual disturbances, whereas neuroticism was associated with a greater burden of postoperative dysphotopsias and showed the strongest association with poorer QoV. At the level of individual visual symptoms, photic phenomena (glare, halos, and starburst), blurred vision, and fluctuation in vision were most consistently associated with personality traits, with the strongest individual correlation observed between neuroticism and starburst perception.

One of the principal findings of the present study is the strong association between neuroticism and postoperative visual symptoms. Neuroticism demonstrated the strongest correlation with global QoV and was positively associated with multiple visual phenomena, including glare, halos, starbursts, hazy vision, blurred vision, double vision, fluctuation in vision, and depth perception difficulties.

These findings are consistent with previous studies investigating the relationship between personality and outcomes following multifocal IOL implantation. A recent systematic review by Pinheiro et al. identified conscientiousness and neuroticism as the personality traits most consistently associated with postoperative satisfaction after multifocal IOL surgery [13]. In their original prospective study, the same group subsequently reported that lower conscientiousness and extraversion were associated with a greater frequency of blurred vision, double vision, and focusing difficulties, whereas higher neuroticism was specifically associated with greater focusing difficulties, following bilateral multifocal IOL implantation [26]. Mester et al. demonstrated that personality traits, particularly compulsive checking, orderliness, competence, and dutifulness, were associated with greater subjective disturbance from glare and halos, highlighting the contribution of personality to postoperative patient satisfaction [18]. Similarly, Rudalevičius et al. reported that patients with predominant neuroticism were the least satisfied with postoperative outcomes, whereas conscientiousness and agreeableness were associated with the highest levels of postoperative satisfaction [19].

Several mechanisms may explain these findings. Individuals with high neuroticism scores typically exhibit greater sensitivity to negative experiences, increased attention to bodily symptoms, and a lower threshold for symptom reporting [16,17]. Neuroticism has also been linked to somatosensory amplification, a construct originally described by Barsky et al. [17], in which normal bodily sensations are perceived as more intense and threatening, which may contribute to increased awareness of postoperative visual disturbances. Consequently, patients with higher neuroticism may perceive dysphotopsias more intensely, assign greater importance to these visual phenomena, or adapt more slowly to postoperative optical changes. The particularly strong association observed with starburst perception may reflect the highly prominent nature of this symptom, especially under mesopic conditions such as night driving [27]. This is consistent with prior reports using the same trifocal IOL platform, in which starbursts were the most frequently reported and most bothersome visual disturbance among patients [4].

In contrast, higher levels of extraversion, agreeableness, conscientiousness, and openness to experience were consistently associated with better subjective quality of vision and fewer postoperative visual complaints. Despite representing distinct behavioral characteristics, these four traits are all linked to greater psychological resilience and more adaptive coping strategies [13,14], which may help patients adjust to postoperative visual phenomena. The optimism and positive affect typically associated with extraversion, for instance, are established predictors of better health-related quality of life and treatment satisfaction [28]. This may explain why extraversion was associated with lower scores across several dysphotopsia and visual-quality domains, and a comparable tolerance for minor visual imperfections could explain the similar pattern seen for agreeableness [28]. Conscientiousness showed the broadest range of significant associations in the present study, consistent with its known links to adaptive health behaviors, treatment adherence, and effective coping [29,30]. Openness to experience, characterized by cognitive flexibility and a willingness to embrace novel experiences [31], may similarly have helped patients accept unfamiliar postoperative visual sensations.

The strong intercorrelations observed among extraversion, agreeableness, conscientiousness, and openness to experience indicate that these traits may not represent fully independent predictors of QoV, but rather partially overlapping expressions of a broader disposition toward psychological adjustment. This finding reinforces the exploratory nature of the trait-specific associations reported here and should temper any interpretation of these four traits as distinct, independent mechanisms. These associations also persisted after adjusting for sex and postoperative visual outcomes, providing further evidence against confounding by these clinical and demographic factors, although the modest sample size limits how many covariates could be evaluated simultaneously.

Neuroadaptation is believed to play a major role in patient satisfaction following multifocal IOL implantation [32,33]. Although its underlying mechanisms remain incompletely understood, successful neuroadaptation enables patients to better tolerate unwanted optical phenomena over time [33]. The present findings raise the possibility that personality traits may influence this adaptive process; however, neuroadaptation itself was not directly assessed in this study, and this hypothesis therefore requires direct testing in future work. Personality-related differences may nonetheless help explain why patients with similar objective visual outcomes experience markedly different levels of postoperative satisfaction.

The finding that age was weakly associated with conscientiousness and neuroticism is consistent with previously reported age-related personality trends [15]. Importantly, this association did not confound the observed personality–QoV relationships, consistent with the view that age may function as a surrogate marker for other underlying factors rather than an independent predictor of postoperative adaptation [27].

These findings have important clinical implications. Personality assessment should not be viewed as a tool for excluding patients from multifocal IOL implantation but rather as an additional component of individualized preoperative counseling [34,35]. Postoperative dissatisfaction should nonetheless first prompt a thorough evaluation for objective clinical causes rather than being attributed to personality alone. Identifying patients with personality profiles associated with lower postoperative satisfaction may help ophthalmologists provide more tailored counseling, establish realistic expectations, and optimize postoperative support when appropriate. These observations should be regarded as preliminary until confirmed in independent studies.

Personality–outcome research of this kind is well established in general medicine. In the broader literature, personality traits—even when accurately measured—typically have limited power to predict how an individual patient will behave or respond [36]. Neuroticism’s association with poorer QoV likely represents one contributing factor rather than the primary explanation for postoperative dissatisfaction.

This study has several limitations. The modest sample size and single-center design may limit how generally these findings apply. However, with *n* = 66 and a two-tailed α of 0.05, this study had approximately 80% power to detect a correlation of |ρ| ≥ 0.34, which was met by four of the five primary personality–QoV correlations; the correlation for openness to experience corresponded to somewhat lower power (~74%). Furthermore, all patients received the same trifocal IOL model, and the observed associations may therefore not be directly applicable to other multifocal or presbyopia-correcting IOL designs. The global QoV score was calculated by summing frequency, severity, and bothersome ratings, assuming equal weighting across items, rather than using the questionnaire’s original Rasch-based subscale scoring. It is possible that excluding patients with complications or suboptimal outcomes introduced some degree of selection bias. Preoperative QoV was not measured, so the reported associations reflect postoperative QoV level rather than change from baseline. Because both personality and QoV were self-reported by the same patients, shared method variance cannot be ruled out as a contributor to the observed associations. Nevertheless, the preoperative assessment of personality traits, together with the standardized surgical and evaluation protocols, represents an important methodological strength. Larger multicenter studies including different IOL technologies are needed to confirm these findings.

## 5. Conclusions

Personality traits are significantly associated with subjective quality of vision following bilateral implantation of a trifocal intraocular lens. Higher neuroticism is associated with poorer quality of vision and increased perception of postoperative dysphotopsias, whereas higher levels of extraversion, agreeableness, conscientiousness, and openness to experience are associated with fewer subjective visual complaints. These findings suggest that psychological characteristics contribute to patient-reported visual outcomes following refractive cataract surgery and may have a role in optimizing preoperative counseling and expectation management for patients considering premium intraocular lens implantation.

## Figures and Tables

**Table 1:** Associations between personality traits and global quality of vision six months after bilateral trifocal IOL implantation.

Personality Trait	Spearman ρ	95% CI	*p*-Value	pFDR
Extraversion	−0.343	−0.541 to −0.111	0.005	0.009
Agreeableness	−0.378	−0.568 to −0.150	0.002	0.006
Conscientiousness	−0.344	−0.541 to −0.111	0.005	0.009
Neuroticism	0.428	0.208 to 0.607	<0.001	<0.005
Openness to experience	−0.318	−0.520 to −0.082	0.009	0.015

*Data are presented as Spearman rank correlation coefficients (ρ) with corresponding 95% confidence intervals (CI). p-values were adjusted for multiple comparisons using the Benjamini–Hochberg false discovery rate (FDR) procedure.*

**Table 2:** Associations between personality traits and individual postoperative visual symptoms.

Visual Symptom		Extraversion	Agreeableness	Conscientiousness	Neuroticism	Openness
Glare	ρ (*p*)	**−0.348 (0.004)**	**−0.373 (0.002)**	**−0.260 (0.035)**	**0.432 (<0.001)**	**−0.337 (0.006)**
	CI interval	**[−0.544 to −0.115]**	**[−0.564 to −0.144]**	**[−0.472 to −0.019]**	**[0.213 to 0.611]**	**[−0.535 to −0.103]**
	pFDR	**0.009**	**0.006**	**0.047**	**0.002**	**0.011**
Halo	ρ (*p*)	**−0.356 (0.003)**	**−0.421 (<0.001)**	**−0.412 (<0.001)**	**0.441 (<0.001)**	**−0.376 (0.002)**
	CI interval	**[−0.551 to −0.125]**	**[−0.602 to −0.200]**	**[−0.595 to −0.189]**	**[0.223 to 0.617]**	**[−0.567 to −0.147]**
	pFDR	**0.008**	**0.002**	**0.003**	**0.002**	**0.006**
Starburst	ρ (*p*)	**−0.344 (0.005)**	**−0.349 (0.004)**	**−0.313 (0.011)**	**0.603 (<0.001)**	**−0.321 (0.009)**
	CI interval	**[−0.541 to −0.112]**	**[−0.545 to −0.117]**	**[−0.516 to −0.077]**	**[0.422 to 0.737]**	**[−0.522 to −0.086]**
	pFDR	**0.009**	**0.009**	**0.017**	**<0.001**	**0.015**
Hazy vision	ρ (*p*)	**−0.302 (0.014)**	**−0.342 (0.005)**	**−0.360 (0.003)**	**0.293 (0.017)**	**−0.274 (0.026)**
	CI interval	**[−0.507 to −0.065]**	**[−0.539 to −0.109]**	**[−0.554 to −0.129]**	**[0.055 to 0.500]**	**[−0.484 to −0.034]**
	pFDR	**0.021**	**0.010**	**0.008**	**0.025**	**0.038**
Blurred vision	ρ (*p*)	**−0.436 (<0.001)**	**−0.524 (<0.001)**	**−0.316 (0.010)**	**0.376 (0.002)**	**−0.403 (<0.001)**
	CI interval	**[−0.613 to −0.217]**	**[−0.680 to −0.323]**	**[−0.518 to −0.080]**	**[0.148 to 0.567]**	**[−0.588 to −0.178]**
	pFDR	**0.002**	**<0.001**	**0.016**	**0.006**	**0.003**
Distortion	ρ (*p*)	−0.096 (0.443)	−0.077 (0.539)	**−0.270 (0.028)**	0.178 (0.152)	−0.085 (0.497)
	CI interval	[−0.330 to 0.150]	[−0.313 to 0.168]	**[−0.481 to −0.030]**	[−0.067 to 0.403]	[−0.321 to 0.160]
	pFDR	0.472	0.550	**0.040**	0.181	0.517
Double vision	ρ (*p*)	−0.211 (0.089)	−0.145 (0.244)	**−0.349 (0.004)**	**0.415 (<0.001)**	−0.169 (0.176)
	CI interval	[−0.431 to 0.032]	[−0.374 to 0.100]	**[−0.545 to −0.117]**	**[0.192 to 0.597]**	[−0.395 to 0.076]
	pFDR	0.114	0.277	**0.009**	**0.003**	0.204
Fluctuation in vision	ρ (*p*)	**−0.430 (<0.001)**	**−0.462 (<0.001)**	**−0.344 (0.005)**	**0.518 (<0.001)**	**−0.328 (0.007)**
	CI interval	**[−0.608 to −0.209]**	**[−0.633 to −0.248]**	**[−0.541 to −0.112]**	**[0.315 to 0.675]**	**[−0.528 to −0.093]**
	pFDR	**0.002**	**0.001**	**0.009**	**<0.001**	**0.013**
Focusing difficulties	ρ (*p*)	−0.134 (0.283)	−0.196 (0.115)	−0.202 (0.103)	0.133 (0.289)	−0.016 (0.896)
	CI interval	[−0.364 to 0.112]	[−0.418 to 0.048]	[−0.424 to 0.042]	[−0.113 to 0.363]	[−0.257 to 0.226]
	pFDR	0.314	0.140	0.129	0.314	0.896
Depth perception	ρ (*p*)	**−0.363 (0.003)**	**−0.381 (0.002)**	**−0.387 (0.001)**	**0.265 (0.031)**	−0.218 (0.078)
	CI interval	**[−0.556 to −0.132]**	**[−0.570 to −0.153]**	**[−0.575 to −0.160]**	**[0.025 to 0.477]**	[−0.437 to 0.025]
	pFDR	**0.008**	**0.006**	**0.005**	**0.043**	0.103

Data are presented as Spearman rank correlation coefficients (ρ) with unadjusted *p*-values shown in parentheses, 95% confidence intervals shown in brackets, and exact Benjamini–Hochberg false discovery rate (FDR)-adjusted *p*-values (pFDR) shown for each association. Bold values indicate associations that remained statistically significant (pFDR < 0.05) after adjustment for multiple comparisons.

## Data Availability

TThe original contributions presented in this study are included in the article. Further inquiries can be directed to the corresponding author.
